# Elucidation of Olive Oil Oxidation Mechanisms by Analysis of Triacylglycerol Hydroperoxide Isomers Using LC-MS/MS

**DOI:** 10.3390/molecules27165282

**Published:** 2022-08-18

**Authors:** Hayato Takahashi, Shunji Kato, Naoki Shimizu, Yurika Otoki, Junya Ito, Masayoshi Sakaino, Takashi Sano, Jun Imagi, Kiyotaka Nakagawa

**Affiliations:** 1 Laboratory of Food Function Analysis, Graduate School of Agricultural Science, Tohoku University, Sendai 980-8572, Miyagi, Japan; 2 J-Oil Mills Innovation Laboratory, Graduate School of Agricultural Science, Tohoku University, Sendai 980-8572, Miyagi, Japan; 3 Food Design Center, J-OIL MILLS, Inc., Yokohama 230-0053, Kanagawa, Japan

**Keywords:** radical oxidation, singlet oxygen oxidation, olive oil, triacylglycerol hydroperoxide, mass spectrometry, oxidation mechanisms, hydroperoxide positional isomers

## Abstract

Despite the importance of the insight about the oxidation mechanisms (i.e., radical and singlet oxygen (^1^O_2_) oxidation) in extra virgin olive oil (EVOO), the elucidation has been difficult due to its various triacylglycerol molecular species and complex matrix. This study tried to evaluate the mechanisms responsible for EVOO oxidation in our daily use by quantitative determination of triacylglycerol hydroperoxide (TGOOH) isomers using LC-MS/MS. The standards of dioleoyl-(hydroperoxy octadecadienoyl)-triacylglycerol and dioleoyl-(hydroperoxy octadecamonoenoyl)-triacylglycerol, which are the predominant TGOOHs contained in EVOO, were prepared. Subsequently, fresh, thermal-, and photo-oxidized EVOO were analyzed. The obtained results mostly agreed with the previously reported characteristics of the radical and ^1^O_2_ oxidation of linoleic acid and oleic acid. This suggests that the methods described in this paper should be valuable in understanding how different factors that determine the quality of EVOO (e.g., olive species, cultivation area, cultivation timing, and extraction methods) contribute to its oxidative stability.

## 1. Introduction

Extra virgin olive oil (EVOO) is rich in monounsaturated fatty acids and contains characteristic components, such as phenolics, tocopherol, carotenoids, and chlorophyll [1,2,3,4,5,6,7,8,9,10,11,12,13]. These components contribute to its unique characteristics (e.g., taste, color, and flavor) and, accordingly, EVOO is used widely in the food and cosmetics industries. However, these characteristics are often impaired by oxidation. Therefore, to provide guidance on preventing oxidation, extensive efforts have been made to reveal the oxidation mechanisms in EVOO [1,2,3,4,5,6,7,8,9,10,11,12,13,14]. Nevertheless, the mechanisms that contribute to the oxidation of EVOO in our daily use are not yet fully understood.

EVOO is mainly composed of linoleic acid (FA 18:2(9*Z*,12*Z*)) and oleic acid (FA 18:1(9*Z*)). These fatty acids can be oxidized by radical oxidation (e.g., auto- and thermal-oxidation) and/or singlet oxygen (^1^O_2_) oxidation (e.g., photo-oxidation) during our daily use [15,16,17]. Radical oxidation is initiated by the abstraction of H· from an allylic hydrogen within the fatty acid structure. It is well-known that FA 18:1(9*Z*) is more resistant to radical oxidation than FA 18:2(9*Z*,12*Z*) due to the absence of a bis-allylic hydrogen. Alternatively, in a typical ^1^O_2_ oxidation reaction, the irradiation of light to photosensitizers yields ^1^O_2_, which reacts with the double bond of fatty acids via the ene reaction. Hence, in ^1^O_2_ oxidation, the rate of hydroperoxide formation is proportional to the number of double bonds [18]. Unlike other edible oils, EVOO contains a high amount of chlorophyll (which acts both as a photosensitizer [1,4,8,19] and a radical scavenger [7,9,20]), various polyphenols [4,7,9] and tocopherols [4,7,9,17,21] (which act as radical scavengers) and carotenoids [4,17,22] (which act as ^1^O_2_ quenchers). Consequently, this complex composition hinders the identification of the mechanisms (i.e., radical or ^1^O_2_ oxidation) that oxidize EVOO in our daily use.

**Figure 1:**
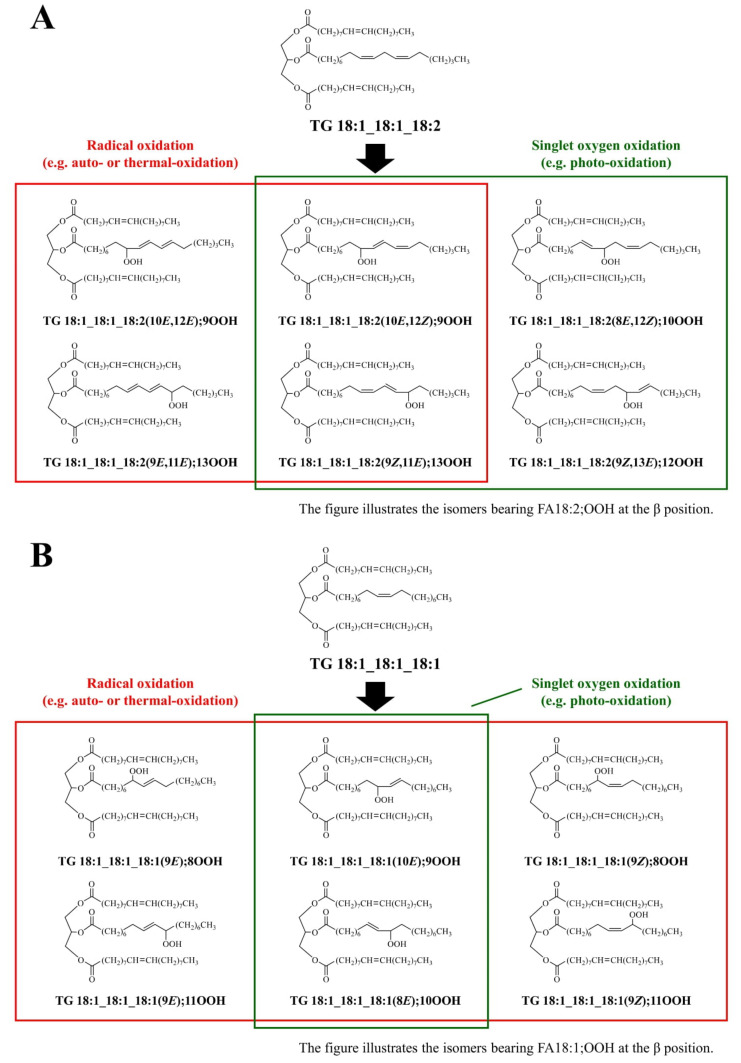
Triacylglycerol (TG) oxidation mechanisms and chemical structures of TG 18:1_18:1_18:2;OOH isomers (**A**) and TG 18:1_18:1_18:1;OOH isomers (**B**). Isomeric structure of TGOOH depends on oxidation mechanisms (radical and ^1^O_2_ oxidation). The shorthand notation of lipids was in accordance with LIPID MAPS [30].

Primary oxidation of lipids affords lipid hydroperoxide (LOOH) isomers, whose structures (i.e., hydroperoxyl group binding positions) depend on oxidation mechanisms (i.e., radical and ^1^O_2_ oxidation; Figure 1) [15,17]. In other words, triacylglycerol (TG) oxidation mechanisms can be identified by characterizing TG hydroperoxide (TGOOH) isomers. However, analyzing TGOOH isomers has been a great challenge, even using the latest analytical techniques and instruments [23,24,25,26,27]. To overcome such issues, we recently developed methods to analyze the positional isomers of various LOOHs by utilizing sodium ions during electrospray ionization (ESI)-LC-MS/MS [28,29]. Using this method, we analyzed the major TGOOH contained in canola oil (TG 18:1_18:1_18:2;OOH) and identified that canola oil was predominantly oxidized by ^1^O_2_ oxidation during storage [29]. The study led us to believe that our LC-MS/MS method can further be applied to elucidate the oxidation mechanisms of EVOO that possess a more complex matrix than canola oil.

In this study, to elucidate the oxidation mechanisms of EVOO, we analyzed TG 18:1_18:1_18:2;OOH isomers in fresh, thermally oxidized, and photo-oxidized EVOO using our LC-MS/MS method described above. Oxidation was performed under conditions that resembled our daily use (e.g., storage and cooking). Additionally, because the susceptibility of FA 18:1(9*Z*) towards both radical and ^1^O_2_ oxidation is different from that of FA 18:2(9*Z*,12*Z*), we also aimed to analyze the oxidation mechanisms of FA 18:1(9*Z*). Hence, the predominant TGOOH molecular species in EVOO that possesses FA 18:1;OOH, TG 18:1_18:1_18:1;OOH isomers were also analyzed. The insights obtained in this study should be helpful to comprehensively understand the mechanisms underlying the oxidation of EVOO.

## 2. Materials and Methods

### 2.1. Materials

TG 18:1_18:1_18:1 was purchased from Sigma-Aldrich (St. Louis, MO, USA). 2-Methoxypropene (MxP) and 2,2′-azobis-(4-methoxy-2,4-dimethylvaleronitrile) (MeO-AMVN) were obtained from FUJIFILM Wako Pure Chemical Corporation (Osaka, Japan). Fresh EVOO was collected immediately after production at J-Oil MILLS, Inc. (Tokyo, Japan) and stored under N_2_ gas and shading until use. All other reagents were of the highest grade available.

### 2.2. Preparation of TG 18:1_18:1_18:2;OOH and TG 18:1_18:1_18:1;OOH Standards

Individual standards of the twelve TG 18:1_18:1_18:2;OOH isomers (Figure 1A) were prepared previously [29].

TG 18:1_18:1_18:1;OOH, as a mixture of the isomers (Figure 1B), was prepared as follows. As an initiator of radical oxidation, 10 µL of MeO-AMVN (500 mg/mL in chloroform) was added to 1 g of TG 18:1_18:1_18:1. To obtain a crude mixture containing TG 18:1_18:1_18:1;OOH isomers, chloroform was evaporated under a N_2_ gas stream and TG 18:1_18:1_18:1 was oxidized under heating at 40–50 °C for 20 h. The hydroperoxyl group of TG 18:1_18:1_18:1;OOH was protected with MxP using a method described previously [28,29,31]. The protected TG 18:1_18:1_18:1;OOH was isolated by semi-preparative HPLC (LC-6AD (Shimadzu, Kyoto, Japan)) using an Inertsil ODS-3 column (10 µm, 20 × 250 mm) at 40 °C with a mobile phase consisting of methanol/2-propanol (3:2, *v*/*v*). The flow rate was set at 20 mL/min and the eluent was monitored with a UV-detector (SPD-20A (Shimadzu, Kyoto, Japan)) at 210 nm. The obtained protected TG 18:1_18:1_18:1;OOH was deprotected and purified as described previously [28,29,31]. A portion of the purified TG 18:1_18:1_18:1;OOH was methyl esterified, subjected to gas chromatography (GC) analysis, and intact acyl residues (i.e., FA 18:1) were measured to determine the concentration of TG 18:1_18:1_18:1;OOH [29]. The prepared TG 18:1_18:1_18:1;OOH was dissolved in 2-propanol and stored at −80 °C until use.

The shorthand notations of TGOOH isomers (and other lipids) described in this study follow the LIPID MAPS nomenclature (Table 1) [30].

### 2.3. MS/MS and LC-MS/MS Analysis of TGOOH Isomers

Q1 mass and product ion mass spectra in the TG 18:1_18:1_18:1;OOH isomer mixture were obtained using a 4000 QTRAP mass spectrometer (SCIEX, Tokyo, Japan). Standard TG 18:1_18:1_18:1;OOH was diluted in methanol (0.5 µM) and directly infused into the MS at a flow rate of 10 µL/min. Positive ESI was used as the ion source. MS spectra were obtained in a range of *m*/*z* 100–1000. Analytical parameters were optimized using the Analyst software (ver. 1.6.2, SCIEX, Tokyo, Japan) (Supplementary Materials).

TG 18:1_18:1_18:2;OOH and TG 18:1_18:1_18:1;OOH isomers were analyzed in multiple-reaction-monitoring (MRM) mode. The MRM transitions described in the Supplementary Materials were used. LC-MS/MS analysis was conducted using an ExionLC HPLC system (SCIEX, Tokyo, Japan) equipped with a 4000 QTRAP mass spectrometer. An Inertsil SIL-100A column (5 µm, 2.1 × 250 mm, GL Sciences Inc., Tokyo, Japan) was eluted with hexane/2-propanol/acetic acid (100:0.6:0.5, *v*/*v*/*v*) at 0.2 mL/min (40 °C). A post-column solvent consisting of methanol/2-propanol (1:1, *v*/*v*) containing 0.2 mM sodium acetate was mixed with the eluent at 0.2 mL/min to promote ionization [29]. TG 18:1_18:1_18:2;OOH and TG 18:1_18:1_18:1;OOH isomers were quantitated with external standard curves.

### 2.4. Oxidation of EVOO

Fresh EVOO (400 mL) was thermally oxidized (radical oxidation) in an amber 500 mL glass beaker under gentle stirring. The beaker was heated in an oil bath kept at 150 °C in the dark.

Photo-oxidation (^1^O_2_ oxidation) of fresh EVOO (400 mL) was performed in a clear 500 mL beaker under gentle stirring. The beaker was irradiated with light-emitting diode (LED) light (5000 lux) at 26 ± 1 °C.

Oxidized EVOO samples were collected at 20 min intervals until 240 min (n = 3). Portions of the collected samples were diluted 10,000-fold in hexane and analyzed with LC-MS/MS (10 µL).

## 3. Results and Discussion

### 3.1. Target TGOOH to Determine EVOO Oxidation Mechanisms

Edible oils, such as EVOO, contain various TG molecular species. Moreover, their oxidation results in an even greater number of hydroperoxyl group positional isomers. Therefore, the analysis of TGOOH isomers in edible oils remains challenging. Meanwhile, because different oxidation mechanisms (e.g., radical and ^1^O_2_ oxidation) afford different hydroperoxide isomers [15,16,17], analysis of TGOOH isomers enables the evaluation of oxidation mechanisms. In this study, we focused on the oxidation of FA 18:2(9*Z*,12*Z*) and FA 18:1(9*Z*). Of the TG molecular species that contain these fatty acids, TG 18:1_18:1_18:2 and TG 18:1_18:1_18:1 are the most predominant in olive oil [26]. Hence, we sought to analyze their hydroperoxides (TG 18:1_18:1_18:2;OOH isomers and TG 18:1_18:1_18:1;OOH isomers) contained in EVOO (Figure 1).

As described above, determining hydroperoxyl group positions is pivotal to evaluate the mechanisms responsible for EVOO oxidation. Thus, previous studies analyzed TGOOH hydroperoxyl group positions after derivatization reactions (e.g., reduction, trimethylsilylation, and methyl esterification). However, because the hydroperoxyl group is relatively unstable, artifacts can be formed during derivatization. Therefore, a direct analysis should be favored over derivatization methods. Meanwhile, most of the previous studies that directly analyzed TGOOH depended solely on molecular weight (i.e., intact TG molecular weight + 32 Da) and, hence, their isomers were not analyzed [23,24,25,26,27]. Under these circumstances, we discovered that the collision-induced dissociation (CID) of the sodium adducts of LOOH provide hydroperoxyl group position-specific product ions based on α-cleavage [28,29]. Using this method, we analyzed hydroperoxyl group positions for the main TGOOH in canola oil (TG 18:1_18:1_18:2;OOH isomers) and found that FA 18:2(9*Z*,12*Z*) in canola oil was oxidized predominantly by ^1^O_2_ oxidation during storage [29]. Therefore, in this study, we aimed to apply the above method to determine the oxidation mechanisms of FA 18:2(9*Z*,12*Z*) in EVOO. Additionally, to obtain further insight into the oxidation of EVOO (i.e., oxidation mechanisms of FA 18:1(9*Z*)), TG 18:1_18:1_18:1;OOH isomers in EVOO were also analyzed.

### 3.2. Analysis of TG 18:1_18:1_18:2;OOH Isomers in Fresh, Thermally Oxidized, and Photo-Oxidized EVOO

**Figure 2:**
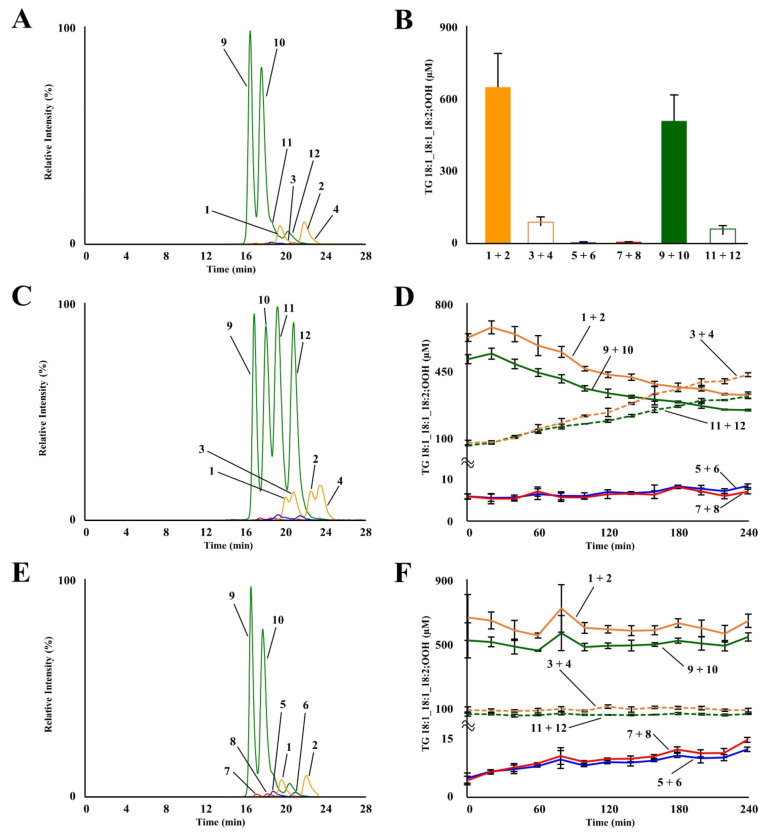
MRM chromatograms of TG 18:1_18:1_18:2;OOH isomers in fresh EVOO (**A**). Refer to Table 1 for peak numbers. Concentration of TG 18:1_18:1_18:2;OOH isomers in fresh EVOO (**B**). MRM chromatograms (**C**) and concentration (**D**) of TG 18:1_18:1_18:2;OOH isomers in thermal-oxidized EVOO. MRM chromatograms (**E**) and concentration (**F**) of TG 18:1_18:1_18:2;OOH isomers in photo-oxidized EVOO. Mean ± SD (n = 3).

TG 18:1_18:1_18:2;OOH isomers in fresh EVOO were analyzed using our previously developed LC-MS/MS method [29]. Typical chromatograms are shown in Figure 2A. Despite being analyzed immediately after opening, TG 18:1_18:1_18:2;OOH isomers were clearly detected from fresh EVOO. Their concentrations were as follows: 647.3 ± 138.4 µM for TG 18:1_18:1_18:2(10*E*,12*Z*);9OOH, 89.1 ± 19.3 µM for TG 18:1_18:1_18:2(10*E*,12*E*);9OOH, 5.6 ± 1.1 µM for TG 18:1_18:1_18:2;10OOH, 5.1 ± 0.9 µM for TG 18:1_18:1_18:2;12OOH, 509.1 ± 105.8 µM for TG 18:1_18:1_18:2(9*Z*,11*E*);13OOH, and 65.3 ± 12.3 µM for TG 18:1_18:1_18:2(9*E*,11*E*);13OOH (Figure 2B). We previously reported that the concentrations of TG 18:1_18:1_18:2;OOH isomers in fresh canola oil were ~0.8 µM [29]. We also identified that ^1^O_2_ oxidation mainly contributed to canola oil oxidation based on isomer analysis [29]. The higher content of these isomers in EVOO, identified in this study, agrees with the fact that EVOO generally has higher peroxide values than other refined oils (e.g., soybean oil and canola oil) [7,8,9,11,12]. EVOO demonstrates higher peroxide values because it is, unlike other oils, typically obtained only by mechanical means and lacks chemical purification processes (e.g., deacidification and deodorizing) to maintain its characteristics (e.g., taste, color, and flavor) [32]. Regarding isomer compositions, concentrations of TG 18:1_18:1_18:2;9OOH and TG 18:1_18:1_18:2;13OOH were higher than those of TG 18:1_18:1_18:2;10OOH and TG 18:1_18:1_18:2;12OOH. Although ^1^O_2_ oxidation of EVOO by chlorophyll is frequently concerned [1,4,8,19], this, interestingly, suggests that ^1^O_2_ oxidation did not significantly contribute to the oxidation of TG 18:1_18:1_18:2.

Subsequently, TG 18:1_18:1_18:2;OOH isomers in thermally oxidized EVOO were analyzed. Thermal oxidation was conducted at 150 °C, the temperature corresponding to a heated pan. Figure 2C represents a typical chromatogram of EVOO heated for 240 min. Concentrations of TG 18:1_18:1_18:2(10*E*,12*E*);9OOH and TG 18:1_18:1_18:2(9*E*,11*E*);13OOH notably increased by thermal oxidation, whereas concentrations of TG 18:1_18:1_18:2(10*E*,12*Z*);9OOH and TG 18:1_18:1_18:2(9*Z*,11*E*);13OOH decreased (Figure 2D). It is well known that the composition of *EZ* and *EE* isomers depends on oxidation temperatures [16,29]. Additionally, studies have shown that the composition of *EZ* and *EE* isomers also depends on the presence of antioxidants because hydroperoxyl radicals isomerize to the thermodynamically favored *EE* form in the absence of proton donors [33]. Therefore, the above composition of *EE* and *EZ* isomers should have reflected the oxidation temperature and the concentration of antioxidants in the EVOO used in this study. Interestingly, the concentration of TG 18:1_18:1_18:2(10*E*,12*E*);9OOH was higher than that of TG 18:1_18:1_18:2(10*E*,12*E*);13OOH, even though their decomposition rates were similar. Meanwhile, in a previous study that investigated the oxidation of FA 18:2(9Z,12Z);1OMe, no difference was observed in the amount of FA 18:2;1OMe,9OOH and FA 18:2;1OMe,13OOH formed [15,17]. Therefore, a preference as to where the hydroperoxyl group is inserted, not seen in the fatty acid form, and may exist in the TG form. Concentrations of TG 18:1_18:1_18:2;10OOH and TG 18:1_18:1_18:2;12OOH did not significantly change during thermal oxidation (Figure 2D).

Photo-oxidation of EVOO was conducted at 5000 lux to represent storage under cloudiness. A typical chromatogram of EVOO, photo-oxidized for 240 min, is shown in Figure 2E. Photo-oxidation of EVOO resulted in a slight increase in the concentrations of TG 18:1_18:1_18:2;10OOH (5.6 ± 1.1 µM (0 min) → 11.8 ± 0.5 µM (240 min)) and TG 18:1_18:1_18:2;12OOH (5.1 ± 0.9 µM (0 min) → 13.8 ± 0.5 µM (240 min)), which are the isomers formed only by ^1^O_2_ oxidation (Figure 2F). ^1^O_2_ oxidation also yields TG 18:1_18:1_18:2(10*E*,12*Z*);9OOH and TG 18:1_18:1_18:2(9*Z*,11*E*);13OOH, in addition to the above two isomers [15,16,17,29]. However, changes in their concentrations were not observed during photo-oxidation, presumably due to their relatively high initial concentrations in the EVOO used in this study.

As we expected, analysis of TG 18:1_18:1_18:2;OOH isomers enabled the elucidation of the oxidation mechanisms of EVOO that possess a complex matrix. On the other hand, the most abundant fatty acid in EVOO is FA 18:1(9*Z*). Therefore, to obtain further insights into the oxidation mechanisms of EVOO, TG 18:1_18:1_18:1;OOH isomers were next analyzed.

### 3.3. MS/MS and LC-MS/MS Analysis of TG 18:1_18:1_18:1;OOH Standards

**Figure 3:**
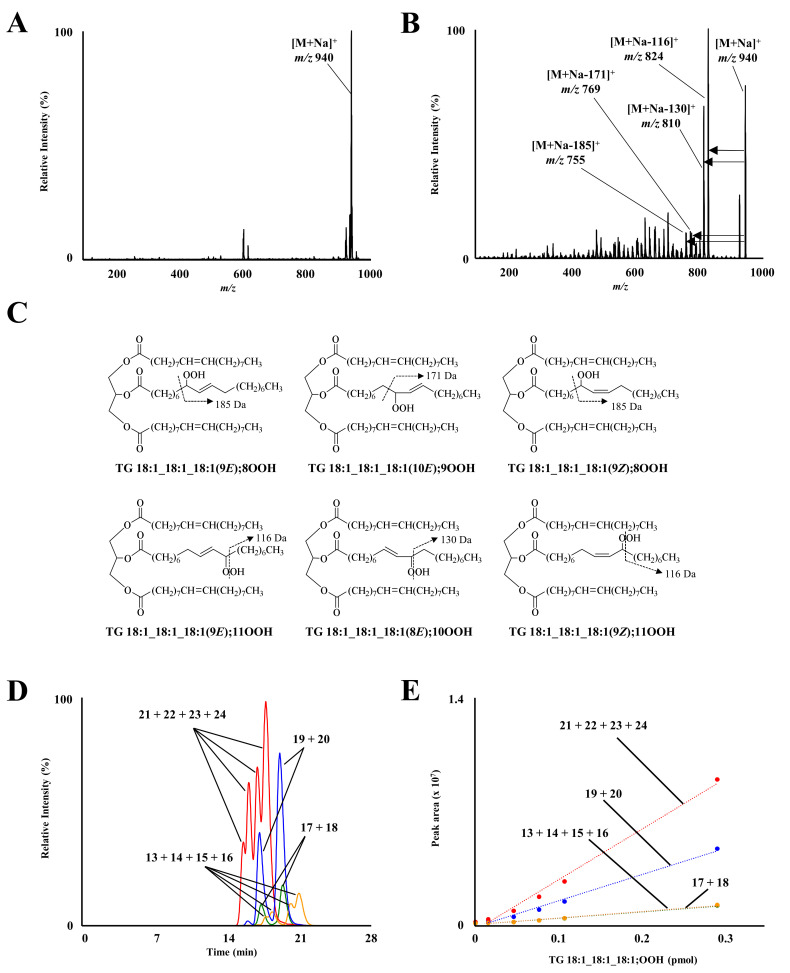
Q1 mass (**A**) and product ion mass (**B**) spectra of prepared TG 18:1_18:1_18:1;OOH isomers. A mixture of TG 18:1_18:1_18:1;OOH isomers (0.5 µM in methanol) was directly infused to the MS/MS system. The ion *m*/*z* 940 [M+Na]^+^ was used as the precursor ion. Proposed fragmentation patterns of TG 18:1_18:1_18:1;OOH isomers (**C**). LC-MS/MS chromatograms of TG 18:1_18:1_18:1;OOH isomers (**D**). A mixture of TG 18:1_18:1_18:1;OOH isomers (0.29 pmol each) were analyzed. Refer to Table 1 for peak numbers. Calibration curves of reference TG 18:1_18:1_18:1;OOH isomers (**E**). Different amounts of TG 18:1_18:1_18:1;OOH isomers (0.015–0.29 pmol) were analyzed by optimized LC-MS/MS. Mean ± SD (n = 3).

Oxidation of TG 18:1_18:1_18:1 yields TG 18:1_18:1_18:1;OOH isomers, whose structures depend on oxidation mechanisms (i.e., radical and ^1^O_2_ oxidation; Figure 1) [15,16,17]. In this study, to accurately analyze TG 18:1_18:1_18:1;OOH isomers and oxidation mechanisms, a standard mixture of the isomers was prepared. We initially attempted to synthesize TG 18:1_18:1_18:1;OOH isomers in a way similar to TG 18:1_18:1_18:2;OOH isomers [29], i.e., by first synthesizing each FA 18:1;OOH isomer, then esterifying them to DG 18:1_18:1. However, because we were unable to separate each FA 18:1;OOH isomer using HPLC, a mixture of TG 18:1_18:1_18:1;OOH isomers was prepared via the direct oxidation of TG 18:1_18:1_18:1. TG 18:1_18:1_18:1 was oxidized with MeO-AMVN, a radical initiator, as all TG 18:1_18:1_18:1;OOH isomers can be formed by radical oxidation [15,16,17]. Then, by selectively protecting the hydroperoxyl group of the crude radical oxidation product with MxP [28,29,31], a mixture of TG 18:1_18:1_18:1;OOH isomers was obtained with high purity. Q1 mass analysis of the prepared standard mixture demonstrated a clear single peak at *m*/*z* 940 ([M+Na]^+^; Figure 3A).

Concentrations of each TGOOH isomer in the prepared mixture were then calculated. Firstly, the total concentration of TG 18:1_18:1_18:1;OOH isomers was determined by GC. Then, we approximated that the mixture contained hydroperoxyl group positional isomers in equal amounts (i.e., TG 18:1_18:1_18:1;8OOH (25%), TG 18:1_18:1_18:1;9OOH (25%), TG 18:1_18:1_18:1;10OOH (25%), and TG 18:1_18:1_18:1;11OOH (25%)). This approximation reflected the radical oxidation pathway of FA 18:1(9*Z*); theoretically, the hydroperoxyl group should be equally distributed among C8–C11 positions. Indeed, a previous study on FA 18:1(9*Z*);1OMe demonstrated that there is barely a preference in hydroperoxide group positions during radical oxidation (i.e., FA 18:1;1OMe,8OOH (26–28%), FA 18:1;1OMe,9OOH (22–25%), FA 18:1;1OMe,10OOH (22–25%), and FA 18:1;1OMe,11OOH (26–28%)) [15,16,17].

Subsequently, product ion analysis of the prepared mixture was performed. Sodium adducts were analyzed based on our previous findings that the CID of LOOH sodium adducts induces hydroperoxyl group position-specific product ions based on α-cleavage [34]. As we expected, each TG 18:1_18:1_18:1;OOH isomer afforded the following specific product ions: *m*/*z* 755 for TG 18:1_18:1_18:1;8OOH, *m*/*z* 769 for TG 18:1_18:1_18:1;9OOH, *m*/*z* 810 for TG 18:1_18:1_18:1;10OOH, and *m*/*z* 824 for TG 18:1_18:1_18:1;11OOH (Figure 3B,C). These product ions were used as MRM transitions (Supplementary Materials) Under optimized LC-MS/MS conditions, clear peaks were detected from the prepared mixture of TG 18:1_18:1_18:1;OOH isomers. MRM of 940 > 755 (TG 18:1_18:1_18:1;8OOH) and 940 > 824 (TG 18:1_18:1_18:1;11OOH) each detected four peaks (Figure 3D), corresponding to the number of fatty acid positional isomers and *EZ* isomers (Figure 1, Table 1). On the other hand, MRM of 940 > 769 (TG 18:1_18:1_18:1;9OOH) and 940 > 810 (TG 18:1_18:1_18:1;10OOH) each detected two peaks corresponding to the number of fatty acid positional isomers (Table 1). Regarding their quantitation, we assumed, based on our previous study [29], that the ionization efficiencies among the fatty acid positional isomers and *EZ* isomers are the same. The prepared calibration curves demonstrated good linearity within a range of 0.0147 to 0.29 pmol for TG 18:1_18:1_18:1;8OOH (r^2^ = 0.9843), TG 18:1_18:1_18:1;9OOH (r^2^ = 0.9889), TG 18:1_18:1_18:1;10OOH (r^2^ = 0.9854), and TG 18:1_18:1_18:1;11OOH (r^2^ = 0.9888) (Figure 3E).

### 3.4. Analysis of TG 18:1_18:1_18:1;OOH Isomers in Fresh, Thermally Oxidized, and Photo-Oxidized EVOO

**Figure 4:**
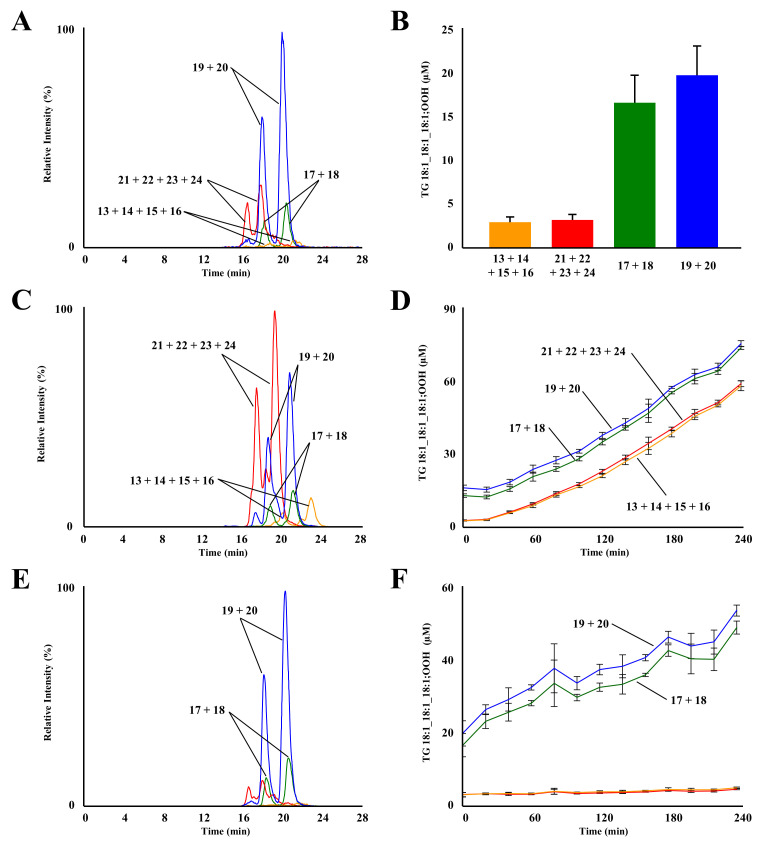
MRM chromatograms of TG 18:1_18:1_18:1;OOH isomers in fresh EVOO (**A**). Refer to Table 1 for peak numbers. Concentration of TG 18:1_18:1_18:1;OOH isomers in fresh EVOO (**B**). MRM chromatograms (**C**) and concentration (**D**) of TG 18:1_18:1_18:1;OOH isomers in thermal-oxidized EVOO. MRM chromatograms (**E**) and concentration (**F**) of TG 18:1_18:1_18:1;OOH isomers in photo-oxidized EVOO. Mean ± SD (n = 3).

TG 18:1_18:1_18:1;OOH isomers were analyzed to determine the oxidation mechanisms of TG 18:1_18:1_18:1 in EVOO (Figure 4A). TG 18:1_18:1_18:1;OOH concentrations in fresh EVOO were as follows: TG 18:1_18:1_18:1;8OOH (3.1 ± 0.6 µM), TG 18:1_18:1_18:1;9OOH (16.5 ± 3.1 µM), TG 18:1_18:1_18:1;10OOH (19.7 ± 3.4 µM), and TG 18:1_18:1_18:1;11OOH (3.1 ± 0.6 µM) (Figure 4B). Concentrations of TG 18:1_18:1_18:1;9OOH and TG 18:1_18:1_18:1;10OOH were notably higher than those of other isomers, suggesting that TG 18:1_18:1_18:1 in fresh EVOO was oxidized mainly by ^1^O_2_ oxidation rather than radical oxidation. These results agreed with the well-known fact that FA 18:1 is tolerant to radical oxidation. Regarding unoxidized TG, the concentration of TG 18:1_18:1_18:1 (567 ± 10 mM) was 2.5-times higher than that of TG 18:1_18:1_18:2 (213 ± 3 mM). However, the concentration of TG 18:1_18:1_18:1;OOH was significantly lower than that of TG 18:1_18:1_18:2;OOH. This suggested that the radical oxidation of TG 18:1_18:1_18:2 was the main factor that contributed to the oxidation of the fresh EVOO analyzed in this study.

Subsequently, TG 18:1_18:1_18:1;OOH isomers in heated EVOO (150 °C) were analyzed. A typical chromatogram of EVOO, thermally oxidized for 240 min, is shown in Figure 4C. Despite the tolerance of FA 18:1 to radical oxidation, all TG 18:1_18:1_18:1;OOH isomers increased with heating time. Isomer compositions followed the radical oxidation pathway of FA 18:1 (Figure 4D); concentrations of each TG 18:1_18:1_18:1;OOH isomer equally increased, as opposed to the case of TG 18:1_18:1_18:2;OOH. This result was consistent with a previous study on the radical oxidation of FA 18:1(9*Z*);1OMe [15,16,17].

In photo-oxidized EVOO (Figure 4E), the concentration of TG 18:1_18:1_18:1;OOH isomers specific to ^1^O_2_ oxidation (i.e., TG 18:1_18:1_18:1;9OOH and TG 18:1_18:1_18:1;10OOH) increased with irradiation time: TG 18:1_18:1_18:1;9OOH (16.5 ± 3.1 µM (0 min) → 48.3 ± 1.8 µM (240 min)) and TG 18:1_18:1_18:1;10OOH (19.7 ± 3.4 µM (0 min) → 52.9 ± 1.5 µM (240 min)) (Figure 4F). These increments were five-times higher than those of TG 18:1_18:1_18:2;10OOH and TG 18:1_18:1_18:2;12OOH (Figure 2F). Considering that the concentration of TG 18:1_18:1_18:1 (567 ± 10 mM) was only 2.5-times higher than that of TG 18:1_18:1_18:2 (213 ± 3 mM), this suggests that the oxidation of FA 18:1(9*Z*) contributes to the photo-oxidation of EVOO. However, ^1^O_2_ oxidation of TG 18:1_18:1_18:2 also affords endoperoxy-hydroperoxides [16,35] and TG 18:1_18:1;OOH_18:2 that were not analyzed in this study. Hence, the analysis of such hydroperoxides should provide further insights into the ^1^O_2_ oxidation of EVOO.

In summary, TG 18:1_18:1_18:2;OOH and TG 18:1_18:1_18:1;OOH isomers were directly analyzed from fresh, thermally oxidized, and photo-oxidized EVOO. To the best of our knowledge, this is the first study reporting analysis of TG 18:1_18:1_18:1;OOH isomers. The obtained results, in most cases, agreed with the previously reported characteristics of the radical and ^1^O_2_ oxidation of FA 18:2(9*Z*,12*Z*) and FA 18:1(9*Z*). Hence, the LC-MS/MS methods reported herein were advantageous in determining oxidation mechanisms of EVOO that possess a complex matrix (i.e., the presence of chlorophyll, tocopherol, various polyphenols, and carotenoids). The methods described in this paper should also be valuable in understanding how different factors that determine the quality of EVOO (e.g., olive species, cultivation area, cultivation timing, and extraction methods) contribute to its oxidative stability.

## Figures and Tables

**Table 1:** The shorthand notation of lipids used in this study was in accordance with LIPID MAPS [30]. The shorthand represents lipid class, constituent fatty acid, geometrical structure, and functional group. For instance, TG 18:1_18:1(*sn*-2)_18:2(10*E*,12*Z*);9OOH means a TGOOH composed of two oleic acids and a 9-hyderoperoxy-10*E*,12*Z*-octadecadienoic acid. The binding position of an oleic acid is defined as *sn*-2 and that of other fatty acids is not defined. Unless otherwise noted, the structures are not defined (e.g., TG 18:1_18:1_18:2(10*E*,12*Z*);9OOH implies both TG 18:1_18:1(*sn*-2)_18:2(10*E*,12*Z*);9OOH and TG 18:1_18:2(10*E*,12*Z*);9OOH(*sn*-2)_18:1. Fatty acids used in this study were FA 18:1(9*Z*) and FA 18:2(9*Z*,12*Z*), and their double bond positions are not mentioned in this paper.

	Molecular Species Level	Hydroperoxyl Group Positional Isomer Level	EZ Isomer Level of Hydrperoxy Fatty Acid	Fatty Acid Positional Isomer Level	Causative Oxidation Mechanism	Compound Number (Figure 2, Figure 3 and Figure 4)
TGOOH	TG 18:1_18:1_18:2;OOH	TG 18:1_18:1_18:2;9OOH	TG 18:1_18:1_18:2(10*E*,12*Z*);9OOH	TG 18:1_18:1(*sn*-2)_18:2(10*E*,12*Z*);9OOH	Radical and ^1^O_2_	**1**
				TG 18:1_18:2(10*E*,12*Z*);9OOH(*sn*-2)_18:1	Radical and ^1^O_2_	**2**
			TG 18:1_18:1_18:2(10*E*,12*E*);9OOH	TG 18:1_18:1(*sn*-2)_18:2(10*E*,12*E*);9OOH	Radical	**3**
				TG 18:1_18:2(10*E*,12*E*);9OOH(*sn*-2)_18:1	Radical	**4**
		TG 18:1_18:1_18:2;10OOH	TG 18:1_18:1_18:2(8*E*,12*Z*);10OOH	TG 18:1_18:1(*sn*-2)_18:2(8*E*,12*Z*);10OOH	^1^O_2_	**5**
				TG 18:1_18:2(8*E*,12*Z*);10OOH(*sn*-2)_18:1	^1^O_2_	**6**
		TG 18:1_18:1_18:2;12OOH	TG 18:1_18:1_18:2(9*Z*,13*E*);12OOH	TG 18:1_18:1(*sn*-2)_18:2(9*Z*,13*E*);12OOH	^1^O_2_	**7**
				TG 18:1_18:2(9*Z*,13*E*);12OOH(*sn*-2)_18:1	^1^O_2_	**8**
		TG 18:1_18:1_18:2;13OOH	TG 18:1_18:1_18:2(9*Z*,11*E*);13OOH	TG 18:1_18:1(*sn*-2)_18:2(9*Z*,11*E*);13OOH	Radical and ^1^O_2_	**9**
				TG 18:1_18:2(9*Z*,11*E*);13OOH(*sn*-2)_18:1	Radical and ^1^O_2_	**10**
			TG 18:1_18:1_18:2(9*E*,11*E*);13OOH	TG 18:1_18:1(*sn*-2)_18:2(9*E*,11*E*);13OOH	Radical	**11**
				TG 18:1_18:2(9*E*,11*E*);13OOH(*sn*-2)_18:1	Radical	**12**
	TG 18:1_18:1_18:1;OOH	TG 18:1_18:1_18:1;8OOH	TG 18:1_18:1_18:1(9*Z*);8OOH	TG 18:1_18:1(*sn*-2)_18:1(9*Z*);8OOH	Radical	**13**
				TG 18:1_18:1(9*Z*);8OOH(s*n*-2)_18:1	Radical	**14**
			TG 18:1_18:1_18:1(9*E*);8OOH	TG 18:1_18:1(*sn*-2)_18:1(9*E*);8OOH	Radical	**15**
				TG 18:1_18:1(9*E*);8OOH(*sn*-2)_18:1	Radical	**16**
		TG 18:1_18:1_18:1;9OOH	TG 18:1_18:1_18:1(10*E*);9OOH	TG 18:1_18:1(*sn*-2)_18:1(10*E*);9OOH	Radical and ^1^O_2_	**17**
				TG 18:1_18:1(10*E*);9OOH(sn-2)_18:1	Radical and ^1^O_2_	**18**
		TG 18:1_18:1_18:1;10OOH	TG 18:1_18:1_18:1(8*E*);10OOH	TG 18:1_18:1(*sn*-2)_18:1(8*E*);10OOH	Radical and ^1^O_2_	**19**
				TG 18:1_18:1(8*E*);10OOH(*sn*-2)_18:1	Radical and ^1^O_2_	**20**
		TG 18:1_18:1_18:1;11OOH	TG 18:1_18:1_18:1(9*Z*);11OOH	TG 18:1_18:1(*sn*-2)_18:1(9*Z*);11OOH	Radical	**21**
				TG 18:1_18:1(9*Z*);11OOH(*sn*-2)_18:1	Radical	**22**
			TG 18:1_18:1_18:1(9*E*);11OOH	TG 18:1_18:1(*sn*-2)_18:1(9*E*);11OOH	Radical	**23**
				TG 18:1_18:1(9*E*);11OOH(*sn*-2)_18:1	Radical	**24**
Fatty Acid Methyl Ester Hydroperoxide	FA 18:2;1OMe,OOH	FA 18:2;1OMe,9OOH	FA 18:2(10*E*,12*Z*);1OMe,9OOH			
			FA 18:2(10*E*,12*E*);1OMe,9OOH			
		FA 18:2;1OMe,13OOH	FA 18:2(9*Z*,11*E*);1OMe,13OOH			
			FA 18:2(9*E*,11*E*);1OMe,13OOH			
	FA 18:1;1OMe,OOH	FA 18:1;1OMe,8OOH	FA 18:1(9*Z*);1OMe,8OOH			
			FA 18:1(9*E*);1OMe,8OOH			
		FA 18:1;1OMe,9OOH	FA 18:1(10*E*);1OMe,9OOH			
		FA 18:1;1OMe,10OOH	FA 18:1(8*E*);1OMe,10OOH			
		FA 18:1;1OMe,11OOH	FA 18:1(9*Z*);1OMe,11OOH			
			FA 18:1(9*E*);1OMe,11OOH			
TG	TG 18:1_18:1_18:2			TG 18:1_18:1(*sn*-2)_18:2		
				TG 18:1_18:1(*sn*-2)_18:2		
	TG 18:1_18:1_18:1					
FA	FA 18:1					
	FA 18:2					

## Data Availability

The datasets used and/or analyzed during the current study are available from the corresponding author on reasonable request.

## Supplementary Materials

The following are available online at https://www.mdpi.com/article/10.3390/molecules27165282/s1, Supplementary Materials: MS and MS/MS parameters for the analysis of TG 18:1_18:1_18:1;OOH and TG 18:1_18:1_18:2;OOH isomers.

## Sample Availability

Samples of the compounds are available from the authors.

## Abbreviations

MeO-AMVN	2,2′-azobis-(4-methoxy-2,4-dimethylvaleronitrile)
CID	collision induced dissociation
ESI	electrospray ionization
EVOO	extra virgin olive oil
LED	light-emitting diode
LOOH	lipid hydroperoxide
MRM	multiple reaction monitoring
MxP	2-methoxypropene
^1^O_2_	singlet oxygen
TG	triacylglycerol
TGOOH	triacylglycerol hydroperoxide
