# Supplementary material for: Elucidation of Olive Oil Oxidation Mechanisms by Analysis of Triacylglycerol Hydroperoxide Isomers Using LC-MS/MS
Source: Molecules. 2022 Aug 18;27(16):5282. doi: 10.3390/molecules27165282 (PMC9415923; doi:10.3390/molecules27165282)
Supplement: Supplementary file 1 [file molecules-27-05282-s001.zip › molecules-1838596-supplementary.pdf]

Supporting Information 1

<table>
<tr><th>Analysis mode</th><th>Q1 mass scan (+)</th><th>Product ion scan (+)</th></tr>
<tr><td>Precursor ion (m/z)</td><td>-</td><td>940</td></tr>
<tr><td>Product ion (m/z)</td><td>-</td><td>-</td></tr>
<tr><td>Scan range (m/z)</td><td colspan="2">100-1000</td></tr>
<tr><td>Curtain gas (psi)</td><td colspan="2">10</td></tr>
<tr><td>Ion spray voltage (V)</td><td colspan="2">5500</td></tr>
<tr><td>Turbo gas temperature (°C)</td><td colspan="2">0</td></tr>
<tr><td>Turbo gas (psi)</td><td colspan="2">15</td></tr>
<tr><td>Nebulizer gas (psi)</td><td colspan="2">0</td></tr>
<tr><td>Collision-activated dissociation gas</td><td>-</td><td>4</td></tr>
<tr><td>Declustering potential (V)</td><td colspan="2">180</td></tr>
<tr><td>Entrance potential (V)</td><td colspan="2">10</td></tr>
<tr><td>Collision energy (V)</td><td>-</td><td>55</td></tr>
<tr><td>Collision cell exit potential (V)</td><td>-</td><td>30</td></tr>
</table>

<table>
<tr><th>Analysis mode</th><th colspan="4">MRM (+)</th></tr>
<tr><td rowspan="2">Precursor ion (m/z)</td><td>TG 18:1_18:1_18:2;9OOH</td><td>TG 18:1_18:1_18:2;10OOH</td><td>TG 18:1_18:1_18:2;12OOH</td><td>TG 18:1_18:1_18:2;13OOH</td></tr>
<tr><td colspan="4">938</td></tr>
<tr><td>Product ion (m/z)</td><td>769</td><td>810</td><td>809</td><td>850</td></tr>
<tr><td>Curtain gas (psi)</td><td colspan="4">20</td></tr>
<tr><td>Ion spray voltage (V)</td><td colspan="4">5500</td></tr>
<tr><td>Turbo gas temperature (°C)</td><td colspan="4">400</td></tr>
<tr><td>Turbo gas (psi)</td><td colspan="4">50</td></tr>
<tr><td>Nebulizer gas (psi)</td><td colspan="4">70</td></tr>
<tr><td>Collision-activated dissociation gas</td><td colspan="4">3</td></tr>
<tr><td>Declustering potential (V)</td><td>66</td><td>66</td><td>51</td><td>66</td></tr>
<tr><td>Entrance potential (V)</td><td>10</td><td>10</td><td>10</td><td>10</td></tr>
<tr><td>Collision energy (V)</td><td>39</td><td>39</td><td>45</td><td>43</td></tr>
<tr><td>Collision cell exit potential (V)</td><td>26</td><td>36</td><td>14</td><td>16</td></tr>
</table>

<table>
<tr><th>Analysis mode</th><th colspan="4">MRM (+)</th></tr>
<tr><td rowspan="2">Precursor ion (m/z)</td><td>TG 18:1_18:1_18:1;8OOH</td><td>TG 18:1_18:1_18:1;9OOH</td><td>TG 18:1_18:1_18:1;10OOH</td><td>TG 18:1_18:1_18:1;11OOH</td></tr>
<tr><td colspan="4">940</td></tr>
<tr><td>Product ion (m/z)</td><td>755</td><td>769</td><td>810</td><td>824</td></tr>
<tr><td>Curtain gas (psi)</td><td colspan="4">20</td></tr>
<tr><td>Ion spray voltage (V)</td><td colspan="4">5500</td></tr>
<tr><td>Turbo gas temperature (°C)</td><td colspan="4">400</td></tr>
<tr><td>Turbo gas (psi)</td><td colspan="4">50</td></tr>
<tr><td>Nebulizer gas (psi)</td><td colspan="4">70</td></tr>
<tr><td>Collision-activated dissociation gas</td><td colspan="4">3</td></tr>
<tr><td>Declustering potential (V)</td><td>180</td><td>176</td><td>186</td><td>180</td></tr>
<tr><td>Entrance potential (V)</td><td>10</td><td>10</td><td>10</td><td>10</td></tr>
<tr><td>Collision energy (V)</td><td>44</td><td>45</td><td>43</td><td>52</td></tr>
<tr><td>Collision cell exit potential (V)</td><td>12</td><td>24</td><td>42</td><td>13</td></tr>
</table>
